# An Improved Binary Differential Evolution Algorithm to Infer Tumor Phylogenetic Trees

**DOI:** 10.1155/2017/5482750

**Published:** 2017-11-27

**Authors:** Ying Liang, Bo Liao, Wen Zhu

**Affiliations:** College of Information Science and Engineering, Hunan University, Changsha, China

## Abstract

Tumourigenesis is a mutation accumulation process, which is likely to start with a mutated founder cell. The evolutionary nature of tumor development makes phylogenetic models suitable for inferring tumor evolution through genetic variation data. Copy number variation (CNV) is the major genetic marker of the genome with more genes, disease loci, and functional elements involved. Fluorescence in situ hybridization (FISH) accurately measures multiple gene copy number of hundreds of single cells. We propose an improved binary differential evolution algorithm, BDEP, to infer tumor phylogenetic tree based on FISH platform. The topology analysis of tumor progression tree shows that the pathway of tumor subcell expansion varies greatly during different stages of tumor formation. And the classification experiment shows that tree-based features are better than data-based features in distinguishing tumor. The constructed phylogenetic trees have great performance in characterizing tumor development process, which outperforms other similar algorithms.

## 1. Introduction

Cancer is the most serious and dangerous disease to human health in the world. Over the past few decades, researchers have been working on the diagnosis and treatment of cancer. Owing to these great efforts, our understanding of cancer has been greatly improved, and early clinical diagnosis and reliable treatment are critical for cancer [1]. Cancer is the result of an imbalance in the cell cycle of the organism. Each cell of the organism contains a complete genome and has great spontaneity [1]. When the genome is no longer regulated by normal tissue and the spontaneity of cells is activated, then cancer develops. Tumor cells succumb to different evolutionary pressures and result in constant replication, growth, invasion, and metastasis [1].

In the early days, Nowell [2] proposed the “clonal evolution” theory that combines evolutionary biology with tumor biology. The model suggests a tumor is most likely to start with a mutated cell. Owing to the expansion of one or more cell subclones, tumor cells show high heterogeneity, which is an important characteristic of tumor development [3]. These tumor cells show significant differences even in the same tissue of the same individual. It has been shown that tumor heterogeneity is evolving along with tumor progression [3]. Tumor heterogeneity has been shown to have a significant impact on the diagnosis and treatment of cancer [3, 4].

Because of the evolutionary nature of tumor development, phylogenetic models were used to infer tumor evolution through genetic variation data [5]. Navin et al. [6] found that a single breast tumor may contain multiple cell subclones, and their chromosome copy numbers vary considerably via single-cell DNA copy number data on CGH platform. The development of next-generation sequencing allows people to infer SNVs and their allele frequencies in heterogeneous tumor cell populations. Because of the huge number of SNVs, inference of a complete tumor progression model to explain the observed data has encountered computational difficulties. Nik-Zainal et al. [7] reconstructs phylogenetic tree from inferred SNV frequencies based on two assumptions: (i) no mutation occurs twice in the course of cancer evolution and (ii) no mutation is ever lost. Strino et al. [8] proposed a linear algebra approach based on the two hypotheses to limit the number of possible trees, which can handle up to 25 SNVs. Detection of clones based on SNV frequency data is necessary for inferring phylogeny. Jiao et al. [9] proposes PhyloSub, a Bayesian nonparametric model, to infer the phylogeny and genotype of the major subclonal lineages represented in the population of cancer cells. Miller et al. [10] proposed a variational Bayesian mixture model to identify the number and genetic composition of subclones by analyzing the variant allele frequencies. Hajirasouliha et al. [11] formulate the problem of constructing the subpopulations of tumor cells from the variant allele frequencies (VAFs) as binary tree partition and present an approximation algorithm to solve the max-BTP problem. El-Kebir et al. [12] formulate the problem of reconstructing the clonal evolution of a tumor using SNV as the VAF factorization problem and derives an integer linear programming solution to the VAF factorization problem. Popic et al. [13] propose LICHeE, a novel method to infer the phylogenetic tree of cancer progression from multiple somatic samples. Because of copy number alterations, loss of heterozygosity (LOH), and normal contamination, the allele frequencies of related SNV need to be corrected [14]. Copy number variation is segment loss or duplication of genome sequence ranging from kilo bases (Kb) to mega bases (Mb) in size, which covers 360 Mb and encompasses hundreds of genes, disease loci, and functional elements [15]. CNVs affect gene expressions in human cell-lines, which also play a major role in cancer [16]. Subramanian et al. [17] develop a novel pipeline for building trees of tumor evolution from the unmixed tumor copy number variations (CNVs) data. Oesper et al. [18] introduce ThetA, an algorithm to infer the most likely collection of genome and its proportions in a sample, and identify subclonal CNVs using high-throughput sequencing data. Ha et al. [19] also present a novel probabilistic model, TITAN, to infer CNA and LOH events while accounting for mixtures of cell populations, thereby estimating the proportion of cells harboring each event. Some tumor progression analysis tools combine VAFs of SNVs and population frequencies of structure variations to reconstruct subclonal composition and tumor evolution. PhyloWGS [20] uses copy number alterations to correct the VAFs of affected SNVs and greatly improves subclonal reconstruction compared to existing methods. As tumor is a heterogeneity system, Jiang et al. [21] propose Canopy to identify cell populations and infer phylogenies using both somatic copy number alterations and single-nucleotide alterations from one or more samples derived from a single patient. Li and Xie [22] propose a software package called PyLOH to deconvolve the mixture of normal and tumor cells using copy number alterations and LOH information. Yu et al. [23] introduce CloneCNA to address normal cell contamination, tumor aneuploidy, and intratumor heterogeneity issues and automatically detect clonal and subclonal somatic copy number alterations from heterogeneous tumor samples. El-Kebir et al. [24] develop SPRUCE to construct phylogenetic trees jointly from SNVs and CNAs, which overcomes complexities in simultaneous analysis of SNVs and CNAs.

The samples of the above studies are mixture of cancer cells and stromal cells; analyzing single cells is the most informative approach to assess the heterogeneity within a tumor [5]. Single-cell analysis is not only one more step towards more-sensitive measurements, but also a decisive jump to a more-fundamental understanding of biology [25]. Navin et al. [26] obtain robust high-resolution copy number profiles by sequencing a single cell and infer about the evolution and spread of cancer by examining multiple cells from the same cancer with the Euclidean metric. Traditionally used Euclidean or correlation distances for tree reconstruction from copy number profiles are ill-suited, owing to the dependent and nonidentical distribution of rearrangement events [5]. Fluorescence in situ hybridization (FISH) is a technique that can be used to count the copy number of DNA probes for specific genes or chromosomal regions in potentially hundreds of individual cells of a tumor. Pennington et al. [27] develop a new method combined with expectation maximization to infer unknown parameters for identifying common tumor progression pathways by taking advantage of information on tumor heterogeneity lost to prior microarray-based approaches on a set of fluorescent in situ hybridization (FISH) data. Chowdhury et al. [28–30] propose a software FISHtrees to build evolutionary trees of single tumors with FISH data. FISHtrees models gain or loss of genetic regions at the scale of single genes, whole chromosomes, or the entire genome, including variable rates for different gain and loss events in tumor evolution [30]. Later, Gertz et al. [31] present FISHtrees 3.0, which implements a ploidy-based tree building method based on mixed integer linear programming. The ploidy-based modeling in FISHtrees 3.0 includes a new formulation of the problem of merging trees for changes of a single gene into trees modeling changes in multiple genes and the ploidy [31]. Here, we propose an improved binary differential evolution algorithm to infer phylogenetic trees (BDEP) using CNV data of cervical cancer and breast cancer. The cervical cancer dataset contains the copy number profiles of four genes, and breast cancer dataset is up to eight genes. Liu et al. [32] show that, on average, each cancer can be explained with around six different marker sets. Tumor phylogenetic tree inference can be treated as minimum Steiner tree problem in directed graph, which is a NP-hard problem. BDEP uses differential individual to search for the best approximate solutions, with the help of individual's difference information and neighborhood optimal information to update. BDEP overcomes the weakness that differential evolution algorithm can only be used in continuous search space with advantages of fast convergence and strong robustness.

## 2. Methods

### 2.1. Problem Definition

**Figure 1:**
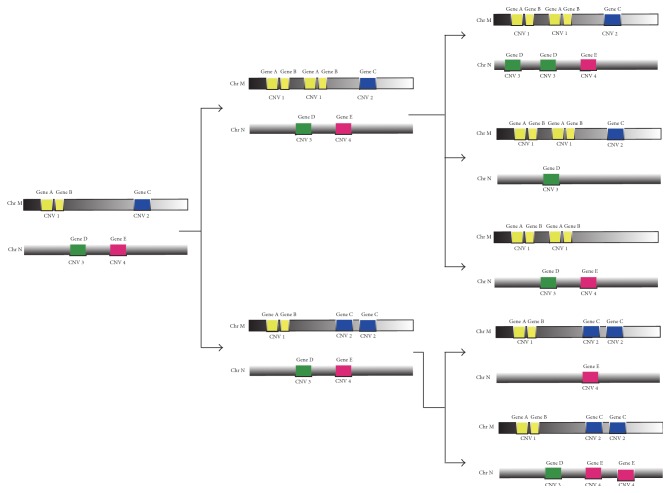
The association between CNVs and genes.

One copy number variation usually affects the copy number of two or more closely related genes [15]. The genes may change their copy number alone or together with their neighbors located in one copy number variation region, which results in computational difficulties of evolution distance between gene copy number profiles (Figure 1). Shamir et al. propose an algorithm that calculates evolution events in linear time and linear space by backtracking the dynamic programming vector [33]. We adopt the idea proposed by Shamir to calculate the minimum variation events between two copy number profiles. Profiles (*u*, *v*) present the evolution distance from the source profile *u* to the target profile *v*. As mentioned by Shamir et al. [33], if the source profile contains the gene with copy number 0 but the target profile with the gene copy number > 0, the transformation from *u* to *v* is unreachable. On the contrary, if the gene has copy number > 0 in the source profile but with the copy number 0 in the target profile, the profiles (*u*, *v*) can be inferred. The distance matrix between copy number profiles is asymmetric, which corresponds to directed edges between copy number profiles.

Cells are continuously growing, proliferating, and dying during the tumor progress; the dying cells disappeared but once played an important role in tumourigenesis. Construct a tree to describe evolutionary relationship of observed cells and dying cells can be regarded as Steiner tree problem; the dying cells in Steiner tree are Steiner node. The Steiner tree problem is a classical combinatorial optimization problem, which has important applications in the fields of computer network layout, circuit design, and biological network analysis. In the paper, the tumor phylogenetic tree is a Steiner minimum tree problem in graph, which is proposed by Hakimi [34] and Hwang et al. [35]. The problem can be described as follows: Given a directed connected graph *G* = (*V*, *E*) with observed nodes and all possible Steiner nodes, *V*, and edges, *E*, each node presents a copy number profile and each edge presents the evolution direction between nodes. The weight of each edge presents the evolution distance between copy number profiles. There is a subset *P*⊆*V*; each element presents the observed copy number profile of cell. The Steiner tree problem is to find a subtree *T* of directed connected graph *G*, which contains all nodes in *P* with minimal weight sum. The subtree *T* is the Steiner tree of subset *P*; the node that exists in *T* but not in *P* is the Steiner node. When *P* = *V*, the Steiner tree problem is minimum arborescence problem, which can be worked out in polynomial time [36]. Otherwise, the Steiner tree problem has no polynomial time solution, which is a NP-hard problem [37]. When the input scale becomes large, it is impossible to find the exact optimal solution in polynomial time. Therefore, a good approximation algorithm will provide a compromise solution for the NP-hard problem.

### 2.2. The Improved Binary Differential Evolution Model

The differential (DE) evolution algorithm does not depend on the characteristics information of problem, with the help of difference information among individuals to disturb the formation of individual and then to search the entire population space. Greedy competition mechanism is employed to seek the optimal solution of the problem. DE algorithm is a population-based stochastic direct search method, which is based on real number coding [38]. The differential evolution algorithm has the advantages of fast convergence, simple operation, easy programming, and strong robustness, which have been widely used in various fields [39–42]. The DE algorithm contains three basic operations: mutation, crossover, and selection. The initial population is randomly generated and covers the entire search space.

*Initial Population*. Suppose *X*_*i*,*G*_ = {*x*_*i*,*G*_^1^,…, *x*_*i*,*G*_^*n*^} is the *i*th individual of generation *G*th; *n* is the dimension of individual; *i* = 1,2,…, *M* is the population scale; *G* = 1,2,…, *G*_max_ is the maximum evolution generation. The initial population of DE is generated by

(1) $$\begin{array}{c} x_{i,0}^{j}=rand_{j}\left(\;0,1\;\right)\left(\;x_{U}^{j}-x_{L}^{j}\;\right)+x_{L}^{j}, \end{array}$$

where *x*_*U*_^*j*^ and *x*_*L*_^*j*^ represent the upper and lower bounds of the *j*th dimension, respectively, and rand_*j*_(0,1) represents a random number within the range [0,1]. 

*Mutation Operation*. Randomly select two different individuals *X*_*p*_1_,*G*_, *X*_*p*_2_,*G*_ to produce the mutant individual *V*_*i*,*G*_ corresponding to individual *X*_*i*,*G*_ as

(2) $$\begin{array}{c} v_{i,G}^{j}=x_{i,G}^{j}+\lambda \left(\;x_{p_{1},G}^{j}-x_{p_{2},G}^{j}\;\right), \end{array}$$

where *x*_*p*_1_,*G*_^*j*^ − *x*_*p*_2_,*G*_^*j*^ is difference vector and scaling factor *λ* is a positive control parameter of difference vector. 

*Crossover Operation*. Crossover operation aims at increasing population diversity. The crossover strategy exchanges mutant and old individual's information to generate trial individual *U*_*i*,*G*_. The crossover operation is defined as

(3) $$\begin{array}{c} u_{i,G}^{j}=\left\{\begin{array}{cc} v_{i,G}^{j} & rand_{j}\left[0,1\right)\leq CR\text{ or }j=rand\left(i\right)\\ x_{i,G}^{j} & \text{otherwise.} \end{array}\right. \end{array}$$

The crossover strategy ensures that *U*_*i*,*G*_ has at least one element from *V*_*i*,*G*_. The crossover rate CR can be adjusted by user within the range [0, 1].

*Selection Operation*. Trial individual *U*_*i*,*G*_ will become a member of the next-generation population, if the fitness function values of *U*_*i*,*G*_ are superior to *X*_*i*,*G*_. Otherwise, the individual *X*_*i*,*G*_ will remain in the next-generation population. The selection operation is defined as

(4) $$\begin{array}{c} X_{i,G+1}=\left\{\begin{array}{cc} U_{i,G}, & \text{fitness}\left(U_{i,G}\right)\leq \text{fitness}\left(X_{i,G}\right)\\ X_{i,G}, & \text{otherwise.} \end{array}\right. \end{array}$$

Perform the above three operations repeatedly until the stopping criterion is satisfied.

#### 2.2.1. Binary Differential Evolution Algorithm

Conventional DE algorithm focuses on the problem of continuous search space, which cannot solve the discrete problem. Also the DE algorithm does not take into account the global or neighborhood optimal individual information. In this paper, we propose a novel binary differential evolution algorithm (BDEP) to solve the Steiner tree problem and further construct tumor phylogenetic tree. In BDEP, trial individual absorbs neighborhood optimal individual information to update at crossover phase. BDEP is different from conventional DE algorithm at initial population operation, mutation operation, and crossover operation. The algorithm flow chart of BDEP is in Algorithm 1. 

*Candidate Steiner Node Generation*. The Steiner tree problem in graph is to find a minimum arborescence which at least contains all nodes in subset *P*. The set of nodes *V* in graph *G* includes the nodes in *P* and all possible Steiner nodes. Before applying Chu-Liu's algorithm to find the minimum arborescence, it is prerequisite to compute all possible Steiner points. The candidate Steiner node is generated according to the gene copy number profile in subset *P*. Under maximum parsimony criterion, the evolutionary distance from gene copy number profile to the candidate Steiner node is 1. As a result, the set of nodes *V* consists of candidate Steiner nodes and subset *P*, which corresponds to a complete directed graph *G*. 

*Individual Encoding*. The individual *i* of binary differential evolution is encoded as a binary string *X*_*i*_ = (*x*_*i*_^1^, *x*_*i*_^2^,…, *x*_*i*_^*n*^), where *x*_*i*_^*j*^ is a binary variable corresponding to the *j*th candidate Steiner node and *n* is the number of candidate Steiner nodes. When *x*_*i*_^*j*^ = 1, the *i*th individual has the *j*th candidate Steiner node. With the gene copy number profile in set *P*, each individual represents a phylogenetic tree; the fitness function is the distance sum of the phylogenetic tree. The objective of BDEP is to find a minimum arborescence representing tumor phylogenetic tree. 

*Initial Population*. The population initialization of BDEP is as follows:

(5) $$\begin{array}{c} x_{i,0}^{j}=\left\{\begin{array}{cc} 1 & rand_{j}\left(0,1\right)<0.05\\ 0 & \text{otherwise.} \end{array}\right. \end{array}$$

The meaning of *i*, *j*, and rand_*j*_(0,1) is the same as that of conventional DE algorithm. 

*Mutation Operation*. For each individual *X*_*i*,*G*_, randomly select two different individuals *X*_*p*_1_,*G*_, *X*_*p*_2_,*G*_ to produce the mutant individual *V*_*i*,*G*_ as follows:

(6) $$\begin{array}{c} v_{i,G}^{j}=\left\{\begin{array}{cc} x_{p_{1},G}^{j}\;|\;x_{p_{2},G}^{j} & x_{p_{1},G}^{j}=x_{p_{2},G}^{j}\\ x_{i,G}^{j} & \text{otherwise.} \end{array}\right. \end{array}$$

For the *j*th candidate Steiner node, if individuals *X*_*p*_1_,*G*_, *X*_*p*_2_,*G*_ have the same choice, the mutant individual yields *x*_*p*_1_,*G*_^*j*^ or *x*_*p*_2_,*G*_^*j*^; otherwise it directly derives from *X*_*i*,*G*_. 

*Crossover Operation*. Social learning is an important way to improve population diversity and self-adaptability. The individual would influence its neighbors: BDEP uses local neighborhood as social learning areas. BDEP adopts the ring topology of population with radius *r* to define local neighborhoods. The *r*-neighborhood of individual *i* is represented as {*R*_*j*_∣|*i* − *j*| ≤ *r*, *j* = 0,1, 2,…, *M* − 1}. The individual *V*_*n*best,*G*_ represents the best neighbors with minimum fitness value in the *r*-neighborhood of mutant individual *V*_*i*,*G*_. The cross operation is according to

(7) $$\begin{array}{c} u_{i,G}^{j}=\left\{\begin{array}{cc} v_{nbest,G}^{j} & rand_{j}\left[0,1\right)\leq CR\text{ or }j=rand\left(i\right)\\ v_{i,G}^{j} & \text{otherwise.} \end{array}\right. \end{array}$$

The crossover strategy exchanges mutant individual and its best neighbor's information to generate trial individual. The crossover rate CR can be adjusted by user within the range [0, 1]. The crossover strategy ensures that *U*_*i*,*G*_ has at least one element from the best neighbor. The neighborhood radius *r* depends on population scale and the complexity of problem. 

*Selection Operation*. The selection strategy is similar to conventional DE algorithm; whether the trial individual *U*_*i*,*G*_ could become a member of the next-generation population depends on fitness function values. If the new individual *U*_*i*,*G*_ is superior to old one *X*_*i*,*G*_, *U*_*i*,*G*_ would replace *X*_*i*,*G*_. Otherwise, the individual *X*_*i*,*G*_ will remain in the next-generation population.

Repeatedly perform the above three operations until one of the two criteria is satisfied: (i) evolutional iterations reach the maximal generation; (ii) the optimal fitness value is less than the distance sum of minimum arborescence of subset *P* and stays unchanged in ten consecutive iterations.

## 3. Results and Discussion

In this section, we apply BDEP to the gene copy number profiles of real tumor and infer the tumor phylogeny of all samples. We study the differences between tumors by statistically analyzing topological features of phylogenetic tree in the following three aspects: branch, level, and edge. And classification experiments are performed to evaluate the merits of these features. The algorithm parameters are set as follows: the max generation *G*_max_ is 100; crossover rate (CR) is 0.7 by default; and population size depends on the complexity of the problem ranging from 300 to 500.

### 3.1. Datasets

Two FISH datasets, cervical cancer and breast cancer, respectively, from Wangsa et al. [43] and Heselmeyer-Haddad et al. [44], are published to visualize copy number changes in tumors based on single-cell analyses. The cervical cancer dataset comprises four probes targeting the genes LAMP3, PROX1, PRKAA1, and CCND1, in pretreatment cervical biopsies from 16 lymph node positive samples and 15 lymph node negative controls from women with stage IB and IIA cervical cancer [43]. The lymph node positive samples contain primary tumors and associated lymph node metastases. The four target genes come from different chromosomes: LAMP3 is a gene located on chromosome 3q26, PROX1 is located on chromosome 1q41, PRKAA1 is located on chromosome 5p19, and CCND1 is located on chromosome 11q13; and altered expression of this gene has been observed in many cancers [43]. The cell number of cervical cancer among 47 cases ranges from 212 to 250 (average cell number is 243), which is not significantly different among primary cancer with positive lymph node, lymph node metastases cases, and lymph node negative controls. But the number of cell gene profiles among them is strikingly different; each gene copy number profile is a tree node in phylogenetic model. The gene profile number of primary cases with positive lymph node ranges from 63 to 187, average being 111. The profile number of lymph node metastases cases ranges from 34 to 115, average being 70. The profile number of lymph node negative controls ranges from 58 to 157, average being 97.

The breast cancer dataset comprises 13 cases of synchronous ductal carcinoma in situ (DCIS) and invasive ductal carcinoma (IDC), which contains eight probes targeting five oncogenes, COX2, MYC, HER2, CCND1, and ZNF217, and three tumor suppressor genes, DBC2, CDH1, and TP53 [44]. The DCIS is considered a precursor lesion for invasive breast cancer, which has a lower degree of chromosomal instability than the IDC [44]. COX2 is located on 1q31.1 and is upregulated in human breast cancer; DBC2 and MYC both are located on chromosome 8; MYC is also upregulated gene in many types of cancers; CDH1 is located on 16q22.1, HER2 and TP53 both are located on chromosome 17, and ZNF217 is located on 20q13.2, which is a strong candidate oncogene for breast and other cancers [44]. The cell number of breast cancer among 26 cases ranges from 76 to 220, average cell number being 142. The cell number and profile number between DCIS and IDC cases are not significantly different. The profile number of DCIS cases ranges from 28 to 143, average being 73. The profile number of IDC cases ranges from 44 to 119, average being 85.

In FISH datasets, gene copy number profiles of each cell are expressed in matrix form, where each row represents a cell case and each column represents a gene probe. The corresponding gene copy number of each cell is a nonnegative integer. The profile with gene copy number of 2 is considered as the root node of tumor evolutionary tree. The datasets can be downloaded at ftp://ftp.ncbi.nlm.nih.gov/pub/FISHtrees/.

### 3.2. Results on Breast Cancer Datasets

**Figure 2:**
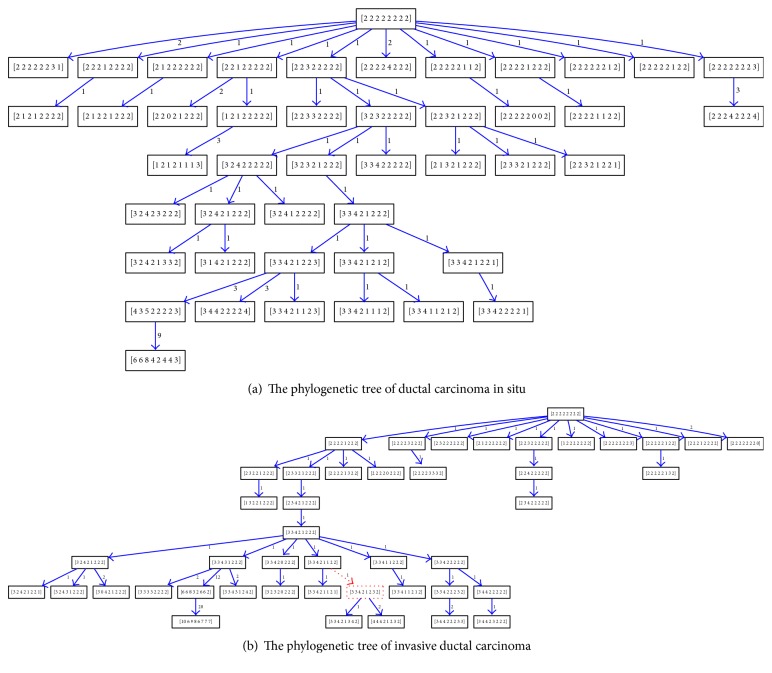
The comparison of BC phylogenetic trees.

We apply BDEP algorithms to the gene copy number profiles of breast cancer and comparatively analyze the tree topology between paired DCIS and IDC samples. We first analyze the branch features of phylogenetic tree at different stages. The branch is defined as subtree derived from the *i*th child of the root node. The DBC2 and MYC gene are on chromosome 8, and TP53 and HER2 gene are on chromosome 17. The copy number of genes lying on the same chromosome is easily affected by CNV simultaneously, phylogenetic trees have at most twenty branches, and we use Chi-square test to compare the distribution characteristics of cell numbers of each branch. The *P* values of Chi-square test from 13 paired samples are listed in Table 1. The *P* value of Chi-square test less than 0.01 is considered significant. For patients 7 and 12, the branch structures of phylogenetic tree are similar. But the branch structures of the remaining 11 paired samples are significantly different, which means that, under different selection pressures, the pathways of tumor subcellular amplification also change. As shown in Figure 2, which is an example of tumor phylogenetic tree from patient 5, Figures 2(a) and 2(b) are, respectively, from DCIS and IDC samples. The node in red is Steiner node and the weight is evolution distance between two nodes. The DCIS phylogenetic tree is more balanced, with more cells concentrated in the first four levels.

**Figure 3:**
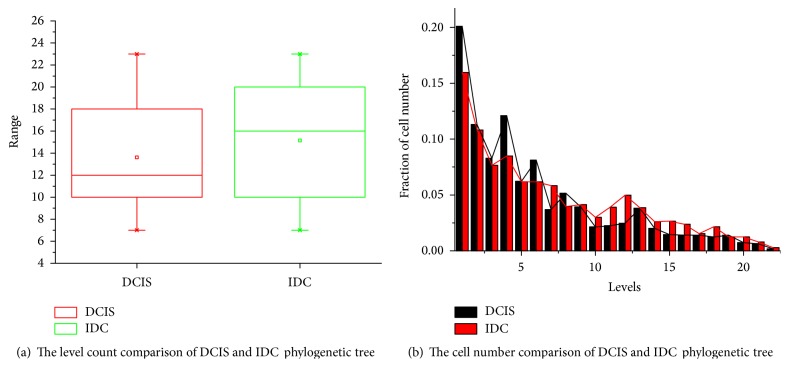
The level characteristics of BC phylogenetic tree.

The cells number of phylogenetic tree across levels between DCIS and IDC tumor shows a noticeable difference. The *P* value of Chi-square test across the first twenty-two levels is listed in Table 1; the root node is on level zero. For the 13 paired samples, there are 11 cases with statistical significance. The hierarchical topology of primary and metastasis trees is similar in patients 3 and 6. We also analyze the depth characteristics of trees and corresponding fraction of cell number at each level. From Figure 3(a), the depth of DCIS tree is not distinctly different from IDC. The cell number distribution across different levels is illustrated in Figure 3(b). For the first six levels, the cell distribution of DCIS is more concentrated with a greater proportion compared with IDC. The cells gather in the first six levels up to 66% in DCIS and 55% in IDC. The number of cells decreases with the increment of tree levels, especially for DCIS. We also compare the edge features of phylogenetic trees; each edge is the corresponding gene gain or loss in the tree topological structure. The *P* value of edge statistics is not significantly different between DCIS and IDC except for patient 6, which is listed in Table 1.

### 3.3. Results on Cervical Cancer Datasets

#### 3.3.1. Statistical Analysis of Tree Feature

**Figure 4:**
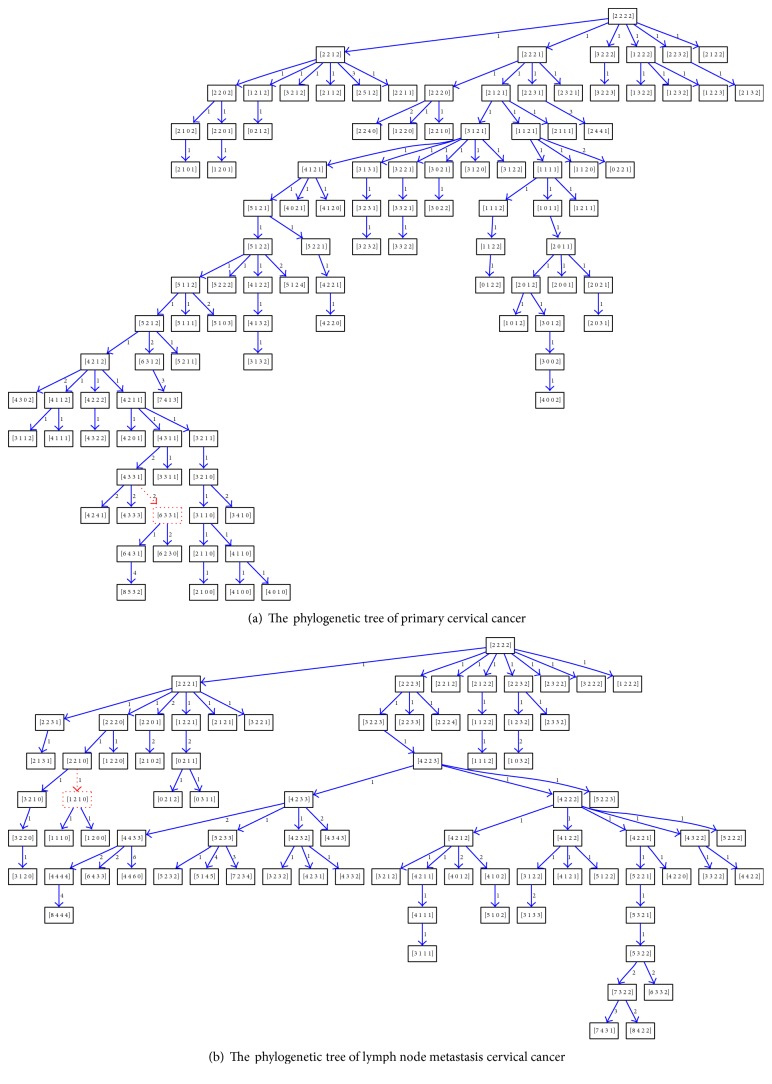
The comparison of CC phylogenetic trees.

BDEP is applied to comparatively analyze the tree topology between paired primary tumor and metastasis samples. The four genes of cervical cancer are on different chromosomes, phylogenetic trees have at most eight branches, and we use Chi-square test to compare the distribution characteristics of cell numbers of each branch. The Chi-square test of branch structure from 16 paired samples shows significant differences, which is listed in Table 2. The tree topology structure of primary and metastasis tumor is quite different. As shown in Figure 4, which is an example of tumor phylogenetic tree from patient 3, Figures 4(a) and 4(b) are, respectively, from primary and metastasis samples. The node in red is Steiner node and the weight is evolution distance between two nodes. The metastasis sample has less copy number profiles, and the corresponding tree has fewer levels but with more balanced and broader topological structure compared with primary one.

In order to find the most decisive gene to distinguish primary and metastasis samples, we analyze the significance of individual gene. For each gene, we compare the cell numbers of branches with gene loss and gain. From Table 2, it is obvious that gene LAMP3 is the most informative gene; there are seven cases showing significant difference (patients 5, 6, 7, 12, 13, 14, and 16), which is consistent with the findings of Kanao et al. [45] and Mine et al. [46]. The overexpression of LAMP3 is associated with an enhanced metastatic potential and may be a prognostic factor for cervical cancer [45]. The gene PRKAA1 is the least with only two significant cases (patients 3 and 11).

**Figure 5:**
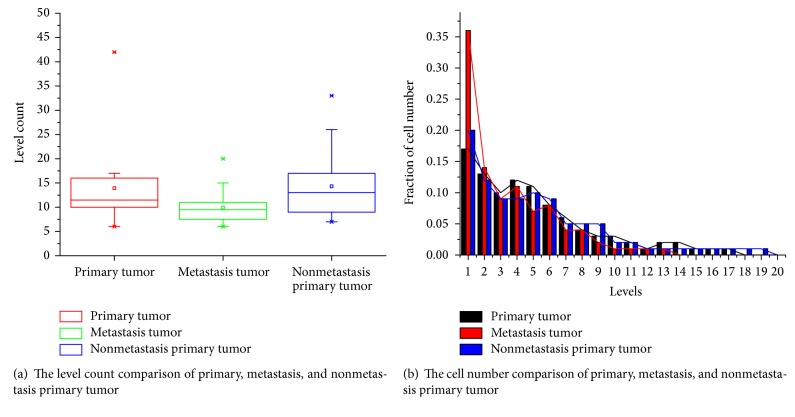
The level characteristics of CC phylogenetic tree.

For the hierarchical structure of trees, the *P* value of Chi-square test across the first twelve levels is listed in Table 3. Among the 16 paired samples, there are 14 cases with statistical significance. The hierarchical topology of primary and metastasis trees is distinguishable except for patients 1 and 9. The depth characteristics of trees and corresponding fraction of cell number at each level are illustrated in Figure 5. Whether or not lymph node later metastasized, the level structure of primary tumor is not distinctly different, but much deeper than the metastasized one. The cell distribution of metastasis sample is more concentrated and most of them gather in the first six levels compared with primary stage tumor. The number of cells decreases with the increment of tree levels, especially for metastasis tumor. The cells gather in the first six levels up to 85% in metastasis tumor and 70% in primary tumor. The cells in primary tumor are more evenly distributed and extending to more levels. For the edge feature of phylogenetic tree, all the 16 paired samples show no significant difference, which is similar to breast cancer samples.

For the edge feature of phylogenetic tree, all the 16 paired samples show no significant difference, which is similar to breast cancer samples.

#### 3.3.2. The Classification Evaluation on Tree Features

The performance to predict the state of the tumor according to topological features of trees is crucial, which provides diagnostic guidance for accurate medical treatment. We evaluate the tree features through classification experiments and compare them with the features directly from data. We use the support vector machines (SVM) as classifier, which is implemented in an open source machine learning Scikit-learn module for Python [47]. We perform three classification experiments on CC dataset and the average accuracy of 100 tests is considered as experimental result. The three classification experiments are as follows:

1. Distinguishing primary from its corresponding metastatic samples, which is a 16 versus 16 samples' classification
2. Distinguishing nonmetastasis primary from primary samples, which is a 15 versus 16 samples' classification
3. Distinguishing primary and nonmetastasis primary samples from metastatic samples, which is a 16 versus 15 versus 16 samples' classification.

**Figure 6:**
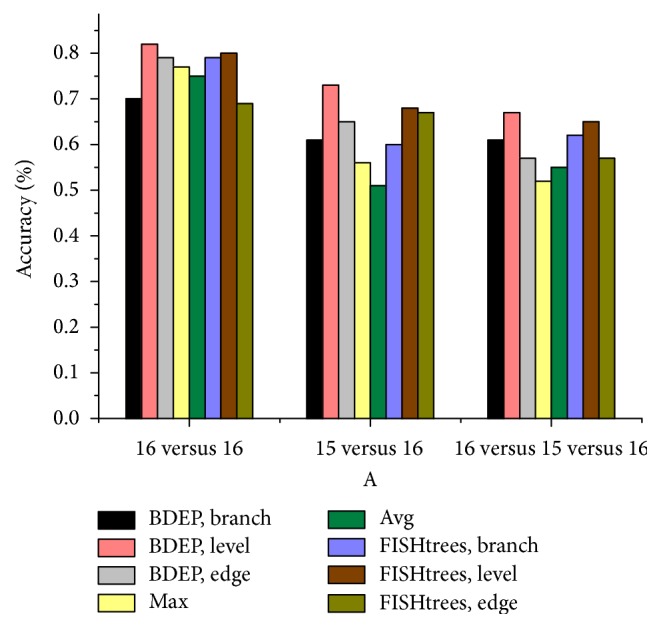
The SVM classification results of different features.

The dataset is divided into four parts: three of them are training sets and the remaining one is test set. The extracted features from tree topology are branch, level, and edge. There are two features derived from data: (i) maximum copy number of each gene; (ii) average copy number of each gene. BDEP also compares with the published FISHtrees algorithm [30], which is a state-of-the-art algorithm for phylogenetic tree based on FISH platform; the result is shown in Figure 6. The experiment distinguishing primary from its corresponding metastatic samples works best, followed by the classification between primary samples. The effect of distinguishing primary, nonmetastasis primary, and metastatic samples is poor for all features. Among all the features, the level feature achieves the highest accuracy, which shows that the degree of cell differentiation varies widely for tumors of different states. The data-based average feature shows in general the worst performance. Also interestingly, the Chi-square tests of branch structure are significant for all 16 paired samples, but classification effect is not as good as expected, even worse than edge feature. FISHtrees works better than BDEP for branch structure feature, but not for edge and level features. Overall, the classification accuracy of tree-based feature is better than data-based feature.

## 4. Conclusion

In this paper, we propose a binary differential evolution algorithm (BDEP) to construct tumor phylogenetic tree via CNV data on FISH platform. Tumor phylogenetic tree inference can be treated as minimum Steiner tree problem in directed graph, which cannot be solved in polynomial time unless no Steiner node exists. The binary differential evolution is a heuristic algorithm with advantages of fast convergence and strong robustness, which provides good approximate solutions with reduced running time. Experimental results on real datasets show that the branch and hierarchical structures have significant differences for tumors of different states. And the gene under different selection pressures would lead to the different pathways of tumor subcellular expansion. The results on classification experiments show that our tree-based features are in general better than data-based features in distinguishing tumor, which provides more accurate and more comprehensive pathological guidance for clinical diagnosis and treatment. The association between genes is the key point to build and understand tumor progression; combining CNV data with other omics data (RNA and DNA methylation) would be a better strategy for tumor phylogenetic tree inference.

## Figures and Tables

**Algorithm 1:**
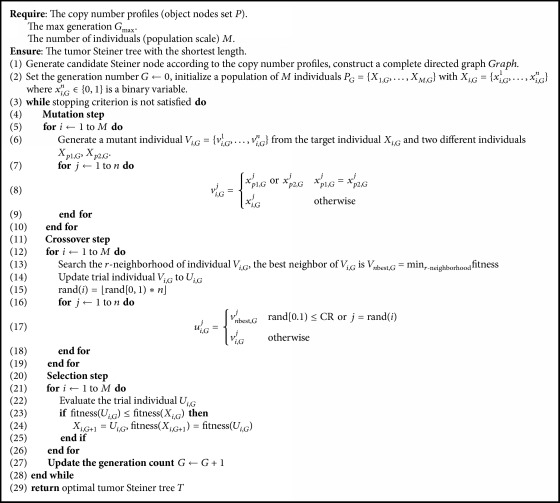
An improved binary differential evolution algorithm to infer tumor phylogenetic trees (BDEP).

**Table 1:** The *P* value of *χ* tests between DCIS and IDC.

Sample ID	*P* value of branches	*P* value of levels	*P* value of edges
Patient 1	4.89*E* − 56	8.40*E* − 03	5.85*E* − 01
Patient 2	4.49*E* − 34	5.61*E* − 20	9.25*E* − 01
Patient 3	1.82*E* − 03	1.38*E* − 02	8.91*E* − 01
Patient 4	5.53*E* − 41	1.86*E* − 06	2.24*E* − 02
Patient 5	2.24*E* − 18	4.28*E* − 03	5.81*E* − 01
Patient 6	4.87*E* − 20	5.22*E* − 02	3.14*E* − 03
Patient 7	6.11*E* − 02	1.06*E* − 05	1.40*E* − 01
Patient 8	2.79*E* − 61	1.45*E* − 20	2.88*E* − 01
Patient 9	1.09*E* − 36	1.50*E* − 18	7.94*E* − 01
Patient 10	6.05*E* − 58	1.38*E* − 11	9.61*E* − 01
Patient 11	1.30*E* − 04	5.96*E* − 16	8.29*E* − 02
Patient 12	7.85*E* − 02	7.40*E* − 06	4.59*E* − 01
Patient 13	2.43*E* − 14	4.01*E* − 05	9.32*E* − 01

**Table 2:** The *P* value of branches *χ* tests between primary and metastasis samples of cervical cancer.

Sample ID	*P* value	*P* value of LAMP3	*P* value of PROX1	*P* value of PRKAA1	*P* value of CCND1
Patient 1	2.56*E* − 15	7.86*E* − 01	3.01*E* − 06	2.16*E* − 01	4.97*E* − 01
Patient 2	6.87*E* − 18	4.05*E* − 02	7.49*E* − 03	9.56*E* − 01	6.32*E* − 01
Patient 3	1.23*E* − 48	8.71*E* − 01	2.90*E* − 01	2.22*E* − 03	3.80*E* − 48
Patient 4	1.00*E* − 48	3.74*E* − 01	1.55*E* − 10	5.24*E* − 02	1.41*E* − 01
Patient 5	1.39*E* − 17	4.65*E* − 05	6.50*E* − 02	5.00*E* − 01	8.74*E* − 01
Patient 6	1.20*E* − 18	3.20*E* − 09	6.01*E* − 02	3.51*E* − 02	4.48*E* − 03
Patient 7	3.64*E* − 28	1.96*E* − 06	5.76*E* − 01	9.55*E* − 01	5.09*E* − 02
Patient 8	8.17*E* − 72	5.47*E* − 01	1.99*E* − 20	1.11*E* − 01	3.45*E* − 03
Patient 9	1.52*E* − 30	6.03*E* − 02	7.52*E* − 02	8.10*E* − 01	9.01*E* − 01
Patient 10	8.15*E* − 10	4.22*E* − 02	6.22*E* − 01	1.44*E* − 01	9.26*E* − 06
Patient 11	1.21*E* − 31	5.65*E* − 01	6.07*E* − 01	1.84*E* − 12	5.63*E* − 05
Patient 12	6.98*E* − 55	1.15*E* − 26	1.41*E* − 06	5.67*E* − 01	7.89*E* − 01
Patient 13	4.71*E* − 73	6.11*E* − 35	9.56*E* − 01	1.89*E* − 02	1.39*E* − 03
Patient 14	2.70*E* − 18	2.29*E* − 06	1.48*E* − 02	5.17*E* − 02	1.20*E* − 02
Patient 15	7.77*E* − 22	6.39*E* − 01	1.72*E* − 03	2.36*E* − 02	3.81*E* − 01
Patient 16	1.19*E* − 27	3.06*E* − 03	8.23*E* − 01	7.50*E* − 01	3.53*E* − 01

**Table 3:** The *P* value of levels and edges *χ* tests between primary and metastasis samples of cervical cancer.

Sample ID	*P* value of levels	*P* value of edges
Patient 1	2.16*E* − 02	9.35*E* − 01
Patient 2	9.81*E* − 09	6.48*E* − 01
Patient 3	3.66*E* − 17	8.04*E* − 01
Patient 4	1.43*E* − 05	9.06*E* − 01
Patient 5	2.79*E* − 07	3.34*E* − 01
Patient 6	6.19*E* − 09	6.82*E* − 01
Patient 7	3.46*E* − 04	9.64*E* − 01
Patient 8	1.22*E* − 07	7.97*E* − 01
Patient 9	1.30*E* − 02	9.25*E* − 01
Patient 10	2.17*E* − 09	8.28*E* − 01
Patient 11	3.84*E* − 10	4.98*E* − 01
Patient 12	1.92*E* − 15	2.49*E* − 01
Patient 13	6.76*E* − 17	2.87*E* − 01
Patient 14	2.34*E* − 06	6.75*E* − 01
Patient 15	7.85*E* − 03	6.48*E* − 01
Patient 16	1.02*E* − 16	9.90*E* − 01
